# Naturally Occurring Resistance-Associated Variants to Hepatitis C Virus Direct-Acting Antiviral Agents in Treatment-Naive HCV Genotype 6a-Infected Patients

**DOI:** 10.1155/2017/9849823

**Published:** 2017-10-15

**Authors:** Zhanyi Li, Ying Liu, Ying Zhang, Xiaoqiong Shao, Qiumin Luo, Xiaoyan Guo, Guoli Lin, Qingxian Cai, Zhixin Zhao, Yutian Chong

**Affiliations:** ^1^Department of Infectious Diseases, Third Affiliated Hospital, Sun Yat-sen University, Guangzhou, Guangdong 510630, China; ^2^Guangdong Provincial Key Laboratory of Liver Disease, Guangdong, China

## Abstract

**Background and Objective:**

The direct-acting antiviral agents (DAAs) antiviral therapy has drastically improved the prognosis of hepatitis C virus (HCV) patients. However, the viral drug resistance-associated variants (RAVs) can limit the efficacy of DAAs. For the HCV-6a is not the predominant prevalent genotype; the data on the prevalence of naturally occurring RAVs in it is scarce. Our study aims to assess the prevalence of RAVs in treatment-naive HCV-6a patients.

**Methods:**

Nested PCR assays were performed on 95 HCV-6a patients to amplify HCV viral regions of NS3, NS5A, and NS5B.

**Results:**

In NS3/4A region, we detected Q80K in 95.5% isolates (84/88) and D168E in 2.3% isolates (2/88). In NS5A region, we detected Q30R in 93.2% isolates (82/88), L31M in 4.6% isolates (4/88), and H58P in 6.8% isolates (6/88). In NS5B region, we detected A15G in 2.3% isolates (2/88), S96T in 1.1% isolates (1/88), and S282T in 20.7% isolates (17/88) and we detected I482L in 100% isolates (4/4), V494A in 50% isolates (2/4), and V499A in 100% isolates (4/4).

**Conclusions:**

RAVs to DAAs preexist in treatment-naive HCV-6a patients. Further studies should address the issue of the impact of RAVs in response to DAA therapies for HCV-6a patients.

## 1. Introduction

Hepatitis C virus (HCV) has infected more than 80 million people (HCV RNA positive) globally. One-third of those who become chronically infected are predicted to develop liver cirrhosis or hepatocellular carcinoma [1]. HCV infection is an important cause of hepatic failure and the liver transplantation of the end stage liver disease [2].

The combination of polyethylene glycol interferon (PEG-IFN) plus ribavirin (RBV) was recommended as the standard of care (SOC) for HCV patients before 2011. However, a sustained virological response (SVR) is only achieved in approximately 50% of patients with HCV genotype (GT) 1 infections [3]. Besides, adverse reactions to these drugs occur in a significant proportion of patients and part of the HCV patients has contraindications before the treatment. More effective and safe treatment was required. Then, scientists discovered some molecules that specifically block various viral proteins [4, 5]. These compounds known as direct-acting antiviral agents (DAAs) are targeted on different viral nonstructural proteins, including the NS3/4A protease, the NS5A protein, and the nucleosides/nonnucleoside NS5B polymerase. Many studies had reported that DAA regimen exhibited a significant advancement in HCV antiviral activity with high SVR rate and insignificant side effects, even in difficult-to-treat patients including old patients, patients with liver cirrhosis, and those in whom PEG-IFN*α*/RBV treatment has failed [6–8]. DAAs have already been recommended to treat HCV-infected patients in combination with PEG-IFN*α*/RBV or in IFN-free regimens as SOC [2, 9, 10].

However, the high replication rate of HCV and the low fidelity of its polymerase combined with selective pressures by the immune system and drug treatment resulted in a sequence variation in the HCV population, leading to a quasispecies and the potential selection of drug resistance-associated variants (RAVs) [11, 12]. Recently, the mutations with varying degrees of drug resistance to DAAs have been detected, even in DAAs-naive patients, and lead to the primary drug resistance [13–16]. These RAVs can affect the efficacy of DAAs because amino acid substitutions within the targeted proteins may affect the viral sensitivity to DAAs [12]. Thus, RAVs are still challenges for the treatment of HCV infection. Because HCV genotype 6a which is frequently seen in Southeast Asia especially in Southern China [17–19] is not the predominant prevalent genotype, previous studies on RAVs are mainly carried out on HCV genotypes 1–4. We face lack of the data on the prevalence of preexisting RAVs in HCV genotype 6a.

None of the DAAs have been approved by the China Food and Drug Administration. In this respect, the use of these drugs appears to be illegal in Mainland China. DAAs may be either unavailable or unaffordable in Mainland China, PEG-IFN*α*/RBV are still the SOC for HCV-infected patients. Most HCV-infected patients were DAAs-naive. However, many types of DAAs have finished the phase III clinical trials. The new era of DAAs in China is dawning. The object of this study was to assess the prevalence of RAVs to DAAs in treatment-naive HCV genotype 6a-infected patients in China.

## 2. Materials and Methods

### 2.1. Patients

817 HCV patients who were admitted into the Third Affiliated Hospital of Sun Yat-sen University between 2009 and 2012 were tested for HCV genotypes, among which 240 cases belong to be HCV genotype 6a. From them, we selected 95 cases who were naïve for antiviral treatment and selected 74 cases of HCV genotype 1b as control. The diagnosis of HCV was based on guidelines on the prevention and treatment of hepatitis C approved by American Association for the Study of Liver Disease. All the patients were Chinese Han population. The work described has been carried out in accordance with The Code of Ethics of the World Medical Association (Declaration of Helsinki) for experiments involving humans. All the study protocols were approved by ethics committee of the Third Affiliated Hospital of Sun Yat-sen University. All patients provided written informed consent.

### 2.2. RNA Extraction, Reverse Transcription, and Quantification

The HCV RNA was extracted from serum samples identified as positive for HCV RNA using 500 *μ*l serum sample and an RNAiso™ Plus extraction kit (Takara Biotechnology Co., Ltd., Dalian, China). The HCV RNA was quantified by detecting the light absorption value using the trace nucleic acid analyzer (Thermo, Carlsbad, CA, USA) at a wavelength of 260 nm. HCV RNA was eluted in 10 *μ*l of Tris-EDTA (TE) buffer and was then reverse transcribed into cDNA using the ReverTra Ace*α*-reverse transcription kit (Toyobo, Shanghai, China), according to the manufacturer's protocol. This cDNA was used as the input for separate PCR assays targeting the HCV core, HCV NS3, HCV NS5A, and HCV NS5B.

### 2.3. HCV Genotyping by Phylogenetic Analysis

HCV core and nonstructural protein 5B (NS5B) regions were amplified using a nested polymerase chain reaction. The primers used for genotyping are listed in Supplemental Table 1, in Supplementary Material available online at https://doi.org/10.1155/2017/9849823. PCR was conducted using the Takara Taq™ PCR kit (Takara Biotechnology Co., Ltd.). The outer PCR system (30 *µ*l) consisted of the following: 3 *µ*l 10x PCR buffer, 2 *µ*l 2.5 mM dNTP, 17.6 *µ*l dH2O, 1.5 *µ*l of each primer (10 pmol/*µ*l), 0.4 *µ*l Taq enzyme (2.5 U/*µ*l), and 4 *µ*l template cDNA. Inner PCR system (30 *µ*l) consisted of the following: 3 *µ*l 10x PCR buffer, 2 *µ*l 2.5 mM dNTP, 19.6 *µ*l dH2O, 1.5 *µ*l of each primer (10 pmol/*µ*l), 0.4 *µ*l Taq enzyme (2.5 U/*µ*l), and 2 *µ*l template cDNA. PCR conditions were as follows: 94°C for 5 min, followed by 30 cycles at 94°C for 30 sec, 55°C for 1 min, and 72°C for 40 sec, and a final step at 72°C for 10 min. DNA was sequenced in both directions using an ABI Prism 3,730 genetic analyzer (Applied Biosystems; Thermo Fisher Scientific, Inc., Waltham, MA, USA).

Then, using the Clustal W 1.8 software package [20] the sequences of HCV strains were aligned with a reference panel of sequences representative of each subtype [21] retrieved from the HCV database (http://talk.ictvonline.org/ictv_wikis/w/sg_flavi/35.table-1-confirmed-hcv-genotypessubtypes-november-2014.aspx). Pairwise distances were generated using the Jukes-Cantor corrected distance algorithm of the program MEGA 5.0 [22]. Phylogenetic analysis was performed using the neighbor-joining method for tree drawing. The reliability of phylogenetic classification was evaluated by a 1,000-cycle bootstrap test.

### 2.4. Amplification and Sequencing of the NS3/4A, NS5A, and NS5B Regions

Specific nested PCR primers for NS3/4A, NS5A, and NS5B regions were designed based on whole genome sequence of subtype 6a isolates (GenBank accession number AY859526, Y12083). The primers are listed in Supplemental Tables 2–4. Due to the high difficulty in amplifying the complete NS5B, the NS5B region was divided into 3 portions based on the characteristics of NS5B region variation loci. The first portion contains A15 and S96. The second portion contains C223, S282, C316, V321, S365, and S368. The third portion contains M414, L419, M423, Y448, I482, and V494. Overlapping primers and seminested PCR were used to increase the amplification success rate. DNA was sequenced in both directions using an ABI Prism 3,730 genetic analyzer (Applied Biosystems; Thermo Fisher Scientific, Inc., Waltham, MA, USA). The sequencing assay was successful in samples containing 1000 IU/mL HCV RNA.

The gene sequence was aligned using the Clustal X program. The NS4A/B, NS5A, and NS5B mutations were analyzed according to the mutations reported in previous studies [23–25]. The comparison was performed according to a subtype 6a isolate (GenBank accession number AY859526). Negative results indicated that the loci had no RAVs when positive results indicated that the loci had RAVs.

### 2.5. Statistical Analysis

SPSS 19.0 (IBM, Armonk, NY, USA) was employed to perform statistical analysis. The clinical characteristics are presented as percentage, or means with standard deviations (SD), or median (minimum, maximum) and two-tailed Student *t*-test, nonparameters, one-AVOVA analysis, and the Mann–Whitney *U* test were adopted to determine the statistical difference, and *P* < 0.05 was considered to be significant.

## 3. Results

### 3.1. Baseline Characteristics of the Patients

The three HCV genes were amplified in 88 of 95 cases. Their mean age was 33.6 ± 14.2 years. 55 patients (62.5%) out of 88 were males and 33 patients (37.5%) were females. Their mean HCV load was 6.9 ± 0.7 (IU/ml log_10_). None of the patients had liver cirrhosis. RVR (rapid virological response), EVR (early virological response), and SVR to the PEG-IFN/RBV treatment were 75.0% (66/88), 78.4% (69/88), and 83.0% (73/88), respectively (Table 1).

### 3.2. Prevalence of RAVs to NS3/4A Protein Protease Inhibitors (PIs)

The success rate of amplification of NS3 was 92.6% (88/95) and the RAVs were present in 100% (88/88) of the isolates. Mutations Q80K and D168E that confer resistance to asunaprevir, paritaprevir, and simeprevir were found in 95.5% (84/88) and 2.3% (2/88) of the isolates, respectively. V36L conferring low-level resistance to telaprevir and boceprevir were found in 4.5% (4/88) of the patients. The frequency of V170I was 98.8% (87/88). However, previous reports indicated that V170I is of unknown clinical relevance (Table 2). In the HCV genotype 1b group, the success rate of amplification of NS3 was 81.08% (60/74). The mutation rate was 38.33% (23/60). Mutations Q80K, D168E, and V36L were not detected in HCV genotype 1b group. Mutations D168Y that confer resistance to asunaprevir, paritaprevir, and simeprevir were found in 1.67% (1/60) of the isolates (Table 2).

### 3.3. Prevalence of RAVs in NS5A

The success rate of amplification of NS5A was 92.6% (88/95) and the prevalence was 100% (88/88). Mutations Q30R and L31M conferring resistance to daclatasvir, ombitasvir, and ledipasvir were found in 82 (93.2%, 82/88) and 4 (4.6%, 4/88) cases, respectively. Mutation H58P associated with resistance to daclatasvir was observed in 6 (6.8%, 6/88) cases. Other RAVs such as M28L, M28F, H54Q, H54T, H58S, H58T, Y93T, and Y93A which were not correlated with clinically relevant resistance were also present at different frequencies (Table 3). In the HCV genotype 1b group, the success rate of amplification was 79.7% (59/74). The mutation rate was 100% (59/59). There were 34 cases (57.6%, 34/59) with Q30R mutation, 1 (1.69%, 1/59) case with L31M mutation, and 51 cases (86.4%, 51/59) with H58P mutation.

### 3.4. Prevalence of RAVS in NS5B

Amplification of NS5B was 92.6% (88/95), 86.3% (82/95), and 4.2% (4/95) for the 5′-end, middle part, and 3′-end, respectively, with 93.2% (69/74), 81.08% (60/74), and 68.92% (51/74), respectively, in the HCV genotype 1b group.

There were 2 (2.3%, 2/88) cases with A15G conferring resistance to PSI-352938 and PSI-353661 with 1 (1.1%, 1/88) case with S96T which associated with resistance to sofosbuvir and mericitabine. Remarkably, 20.7% (17/82) of the cases had the main RAVs S282T which confers resistance to sofosbuvir and mericitabine. We detected 2 cases with I482L + V499A and 2 cases with I482L + V494A + V499A which associated with resistance to tegobuvir, JTK-109, and deleobuvir (Tables 4 and 5). In the HCV genotype 1b group, we detected no case with A15G, S96T, and S282T mutation.

### 3.5. Prevalence of Multiple RAVs

We also found that 87.5% (77/88) of the isolates showed two or more RAVs. There were 53 cases with (NS3-Q80K) + (NS5A-Q30R) and 2 cases with (NS3-Q80K) + (NS5A-Q30R + H58P). There were 13 cases with (NS3-Q80K) + (NS5A-Q30R) + (NS5B-S282T), 2 cases with (NS3-Q80K) + (NS5A-Q30R) + (NS5B-A15G + S282T), 1 case with (NS3-Q80K) + (NS5A-Q30R) + (NS5B-S96T), and 1 case with (NS3-Q80K + D168E) + (NS5A-Q30R) + (NS5B-S282T) with resistance to NS3/4A, NS5A, and NS5B inhibitors such as paritaprevir, daclatasvir, ledipasvir, and sofosbuvir (Table 6). We also found that 85.5% (59/69) of the patients have 2 or more than 2 RAVs which will result in high resistance towards DAAs and resistance to multiple DAAs in the HCV genotype 1b group.

## 4. Discussion

Little data have been published on the natural occurrence of viral variants in HCV genotype 6a; in our study, we investigate the RAVs in DAAs treatment-naive HCV-6a-infected patients. Naturally occurring RAVs may influence virologic response and the efficacy of DAAs-based therapy may be attenuated by the naturally occurring RAVs. It was reported that only 39% of patients with naturally occurring RAVs of NS5A-L31, NS5A-Y93, and NS3-D168 achieved SVR after treatment with daclatasvir and asunaprevir combination therapy, while the SVR rate was 92% in patients without these RAVs [35, 36]. Baseline identification of naturally occurring RAVs in treatment-naïve patients may be helpful for introducing DAAs therapies.

Some RAVs to DAAs were observed in our study. It had been detected that the main sites (R155 and A156) witness less variation, whereas the second sites (V36, T54, Q80, D168, V170) witness variation more frequently. Our study showed that 95.5% (84/88) genotype 6a isolates showed Q80K and 2.3% (2/88) isolates showed D168E which is associated with resistance to PIs such as asunaprevir, paritaprevir, and simeprevir. Paritaprevir combined with ombitasvir, ritonavir, and dasabuvir is IFN-free regimen to treat HCV infections [9]. It is reported that no RAVs to NS3 PIs have been observed in genotype 6 isolates [37] which is in contrast to our finding. This may be due to differences of HCV genotype epidemiology. The prevalence of 95.5% for Q80K for HCV genotype 6a was higher than other genotypes [17, 19, 37]. Q80K may result in high drug resistance which was not detected in genotype 1b isolates in our study. The Q80K variant was associated with different levels of resistance to some approved NS3 PIs (asunaprevir, paritaprevir, and simeprevir). SVR rates in simeprevir-based treatment-naïve HCV genotype 1a infected patients with and without the Q80K variant were 58% versus 84% [25, 27]. NS3/4A PIs may be not suitable for treating HCV genotype 6a patients for the high prevalence of Q80K mutation. Different from the previous studies, main mutations such as R155 and A156 which may result in high drug resistance were not detected in our study when some other variations were found, including V36L and V170I, which have not yet been linked to clinical resistance.

RAVs to NS5A inhibitors are frequently detected as natural variants in HCV genotype 1 infected DAAs-naïve patients. The rate of natural occurrence drug resistance mutations to NS5A inhibitors was estimated at 29.6% by HCV genomic sequencing [38]. In contrast, our study found that the prevalence of naturally occurring RAVs was extremely high; for instance, the prevalence of Q30R, H58P, and L31M was 93.2% (82/88), 6.8% (6/88), and 4.6% (4/88), respectively. The mutation rate of Q30R was 57.63% (34/59) when H58P was 86.44% (51/59) in genotype 1b isolates in our study. We show that the RAV Q30R conferring high levels of resistance to NS5A [13] inhibitors in genotype 1a viruses was prevalent in genotype 6a viruses infecting our Han population. Y93H which confers medium to high level resistance to all three approved NS5A inhibitors (daclatasvir, ombitasvir, and ledipasvir) was not detected in HCV genotype 6a-infected patients and was detected in 5.08% of the genotype 1b isolates. This data seems to be in conflict with the significant prevalence of Y93H in the European and the US HCV genotype 1 isolates (15.0% and 9.3%) [30]. It was reported that 44.4% patients with baseline Y93 or L31 achieved SVR when 89.0% patients without baseline Y93 or L31 achieved SVR after daclatasvir-containing treatment [39]. Dual combinations of mutations confer a higher degree of drug resistance to NS5A PIs such as L31V + Y93H or L31M + Y93H. Cross-resistance is expected between daclatasvir and ledipasvir, mainly due to the presence of mutations at positions L31 and Y93 [40]. Two isolates had dual combinations of mutations L31M + H58P, one patient had Q30R + L31M, and two patients had Q30R + H58P. Their levels of resistance are unknown.

The HCV NS5B is the last nonstructural gene sequence of HCV and is located in the end part genome of the virus. The variation of NS5B amino acid sequence can influence DAAs antiviral capacity and genetic barrier. Main mutations such as S282T which may result in high drug resistance were not detected in genotype 1 isolates in our study. In contrast, we detected 2.3% (2/88) patients had A15G, 1.1% (1/88) patients had S96T, and 20.7% (17/82) patients had S282T which confers resistance to nucleoside/nucleotide analogue NS5B inhibitors, such as PSI-352938, PSI-353661, sofosbuvir, and mericitabine. Sofosbuvir is widely used in DAA-based antiviral therapies and S282T is a major mutation in the NS5B gene which confers high level resistance to sofosbuvir [13]. S282T may result in virologic relapse and sofosbuvir-containing regimens treatment failure [6, 35]. At another aspect, we detected 2 isolates had V494A, 4 patients had I482L, and 4 isolates had V499A which confers resistance to nonnucleoside analogue NS5B inhibitors such as tegobuvir, JTK-109, and deleobuvir.

In addition, we detected that 87.5% isolates harbor one or more RAVs. Chen et al. reported that multiple RAVs were observed, but the frequencies were extremely low [38]. However, Patiño-Galindo et al. reported that, for genotype 6, 67.1% of the sequences presented at least two natural RAVs [41]. Our study showed that multiple RAVs to DAAs were common (87.5%) and even occurred with a higher frequency than the frequency reported by Patiño-Galindo et al. Viruses carrying combinations of RAVs in two or three HCV genes might increase the possibility of failure of combination DAA regimes.

IFN-free regimens were recently recommended for the clinical treatment of HCV infections [2, 9, 10]. They are combined with different types of DAAs, such as NS5A inhibitors and NS5B polymerase inhibitors, NS3/4A protease inhibitors, and NS5B polymerase inhibitors or NS3/4A protease inhibitors, NS5A inhibitors, and NS5B polymerase inhibitors. Whether the multiple RAVs will interfere with the efficacy of IFN-free regimens deserves further studies.

This study had certain limitations. The amplification of the third fragment of NS5B gene was not so successful. Available data to date on RAVs is mainly from the study on HCV genotypes 1–5 and the effect of RAVs in genotype 6a is still not certain.

## 5. Conclusions

In conclusion, RAVs to all three classes of DAAs do exist in untreated HCV-6a-infected patients and their prevalence is high including RAVs associated with clinical resistance to simeprevir, paritaprevir, daclatasvir, ledipasvir, and sofosbuvir. These results may be associated with the different HCV genotype epidemiology in our region. The pattern of the prevalence of RAVs to DAAs is different between HCV-6a-infected patients and HCV-1b-infected patients.

Although the DAAs are not available in China Mainland and the PEG-IFN/RBV therapy is still the SOC for HCV patients, clinicians may consider RAVs as possible challenge for DAA-based antiviral therapies for HCV genotype 6a infection. Resistance testing might help to select the most optimized treatment option. Further studies are needed to find out the impact of naturally present RAVs in response to DAA-based therapies for HCV-6a infected patients.

## Supplementary Material

Primers used for amplifying in our study.

## Figures and Tables

**Table 1:** Characteristics of HCV 6a-infected patients with mutations.

Characteristics	HCV-6a	HCV-1b	*F*/*Z*/*X*^2^	*P*
*N*	88	69		
Age (years)	33.6 ± 14.2	38.5 ± 13.2	0.771	0.033
Male (%)	55 (62.5%)	43 (62.3%)	0.001	1.000
ALT (U/L)	62.5 (10, 425)	54.5 (17, 285)	−0.291	0.772
AST (U/L)	44 (11, 258)	41 (18–164)	−.082	0.414
ALB (g/l)	43.5 ± 3.4	44.7 ± 3.3	0.355	0.054
PLT (×109)	206.3 ± 83.8	192.4 ± 69.0	0.583	0.227
Hb (g/l)	137.6 ± 18.4	147.5 ± 16.7	0.233	0.001
BMI (kg/m2)	21.8 ± 3.9	21.9 ± 2.8	0.039	0.964
HCV RNA (IU/ml log10)	6.9 ± 0.7	7.1 ± 0.7	0.585	0.360
Liver cirrhosis	0	0		
RVR	75.0% (66/88)	65.2% (45/69)	1.787	0.217
EVR	78.4% (69/88)	72.4% (50/69)	0.745	0.454
SVR	83.0% (73/88)	78.3% (54/69)	0.551	0.541

**Table 2:** RAVs to HCV NS3/4A inhibitors.

Resistance mutations	Drugs	References	Detected resistance mutations (HCV 6a *n* = 88)	Detected resistance mutations (HCV 1b *n* = 60)
V36 A/G/ C/L	Boceprevir, paritaprevir, telaprevir	[13, 26]	V36L 4.5% (4/88)	—
T54A/S	Boceprevir, telaprevir	[13, 26, 27]	—	T54S 6.67% (4/60)
V55A	Boceprevir, telaprevir	[13]	—	V55R 1.67% (1/60)
Q80R/K	Asunaprevir, paritaprevir, simeprevir	[13, 14, 28]	Q80K 95.5% (84/88)	Q80L 3.33% (2/60)
R155K/T/Q/I/M/G/L/S	Asunaprevir, boceprevir, paritaprevir, simeprevir, telaprevir	[13, 14, 26]	—	—
A156F/N/S/T/V	Asunaprevir, boceprevir, paritaprevir, simeprevir, telaprevir	[13, 14, 27–29]	—	A156S 18.33% (11/60)
D168G/V/E/H/T/Y	Asunaprevir, Paritaprevir, Simeprevir	[13, 14, 27, 29]	D168E 2.3% (2/88)	D168Y 1.67% (1/60)
V170A	Boceprevir, telaprevir	[13]	V170I 98.8% (87/88)	V170I 15.0% (9/60)
F43I/L/S/V	Asunaprevir, paritaprevir, simeprevir	[14, 19, 27, 29]	—	—
Y56H	Paritaprevir	[13, 14]	—	—
S122R	Asunaprevir, simeprevir	[14]	—	—
V158I	Boceprevir	[13, 26]	—	—
M175L	Boceprevir	[13, 26]	—	—

**Table 3:** RAVs to HCV NS5A inhibitors.

Resistance mutations	Drugs	References	Detected resistance mutations (HCV 6a *n* = 88)	Detected resistance mutations (HCV 1b *n* = 59)
M28T/A/G/V	Daclatasvir, ombitasvir, ledipasvir	[13, 14, 30, 31]	M28L 85.2% (75/88) M28F 13.6% (12/88)	M28L 98.31% (58/59)
Q30E/R/H/L/T	Daclatasvir, ombitasvir, ledipasvir	[13, 14, 30, 31]	Q30R 93.2% (82/88)	Q30R 57.63% (34/59)
L31M/V/I/F	Daclatasvir, ombitasvir, ledipasvir	[13, 30–32]	L31M 4.6% (4/88)	L31M 1.69% (1/59)
H54Y	Daclatasvir	[33]	H54Q 1.1% (1/88) H54T 3.4% (3/88) H54S 1.1% (1/88)	H54Q 83.05% (49/59)
H58P	Daclatasvir	[33]	H58P 6.8% (6/88) H58T 93.2% (82/88)	H58P 86.44% (51/59) H58T 3.39% (2/59) H58S 6.78% (4/59) H58R 3.39% (2/59)
Y93C/N/F/H/S	Daclatasvir, ombitasvir, ledipasvir	[13, 14, 30–33]	Y93A 47.7% (42/88) Y93T 46.6% (41/88)	Y93H 5.08% (3/59) Y93T 1.69% (1/59) Y93A 20.34% (12/59)

**Table 4:** RAVs to nucleoside/nucleotide analogue NS5B inhibitors.

Resistance mutations	Drugs	Reference	Detected resistance mutations	Detected resistance mutations
Case number			*n* = 88	*n* = 69
A15G	PSI-352938 + PSI-353661	[15]	A15G 2.3% (2/88)	—
A15S	PSI-352938 + PSI-353661	[15]	—	—
S96T	Sofosbuvir + mericitabine	[33]	S96T 1.1% (1/88)	—
Case number			*n* = 82	*n* = 60
C223H	Sofosbuvir + mericitabine	[33]	—	—
S282T	Sofosbuvir + mericitabine	[33]	S282T 20.7% (17/82)	—
V321I	PSI-352938 + PSI-353661	[15]	—	—

**Table 5:** RAVs to nonnucleoside NS5B inhibitors.

Resistance mutations	Drugs	Reference	Detected resistance mutations	Detected resistance mutations
Case number			*n* = 82	*N* = 60
C316Y/N/H	Dasabuvir, tegobuvir, HCV796	[13–16, 34]	—	C316N 100% (60/60)
S365T/A	Tegobuvir, HCV796	[15, 16]	S365F 1.2% (1/82) S365P 1.2% (1/82)	S365A 3.33% (2/60)
S368T	Dasabuvir	[14]	S368A 1.2% (1/82) S368L 1.2% (1/82)	—
Case number			*n* = 4	*n* = 51
M414T/I/V/L	Dasabuvir, tegobuvir, HCV796	[13, 15, 16]	M414Q 50% (2/4)	M414L 5.88% (3/51)
L419M/V	Tegobuvir, HCV796	[15, 16]	L419I 100% (4/4)	—
M423T/I/V	Tegobuvir, HCV796	[15, 16]	—	M423I 1.96% (1/51)
Y448C/H	Dasabuvir, tegobuvir	[13–16]	—	Y448H 1.96% (1/51)
I482L/V/T	Tegobuvir	[15, 16]	I482L 100% (4/4)	I482T 5.88% (3/51) I482V 1.96% (1/51)
V494S/Q/L/A/T	Tegobuvir	[15, 16]	V494A 50% (2/4) V494C 25% (1/4)	V494L 3.92% (2/51)
P495S/Q/L/A/T	Tegobuvir	[15, 16]	—	P495S 5.88% (3/51)
P496A/S	Tegobuvir	[15, 16]	—	P496T 5.88% (3/51)
V499A	JTK-109, deleobuvir	[15]	V499A 100% (4/4)	V499A 15.69% (8/51) V499T 1.96% (1/51) V499I 3.92% (2/51)

**Table 6:** Multiple RAVs to DAAs.

Drug resistance mutations	*N*	HCV gene
L31M + H58P	1	NS5A
Q80K + Q30R	53	NS3/4A + NS5A
Q80K + Q30R + L31M	1	NS3/4A + NS5A
L31M + H58P + I482L + V494A + V499A	1	NS5A + NS5B
H58P + I482L + V499A	1	NS5A + NS5B
Q80K + Q30R + A15G + S282T	2	NS3/4A + NS5A + NS5B
Q80K + Q30R + H58P	2	NS3/4A + NS5A + NS5B
Q80K + Q30R + S282T	13	NS3/4A + NS5A + NS5B
Q80K + Q30R + S96T	1	NS3/4A + NS5A + NS5B
Q80K + D168E + Q30R + S282T	1	NS3/4A + NS5A + NS5B
Q80K + Q30R + I482L + V499A	1	NS3/4A + NS5A + NS5B
