# Supplementary material for: Naturally Occurring Resistance-Associated Variants to Hepatitis C Virus Direct-Acting Antiviral Agents in Treatment-Naive HCV Genotype 6a-Infected Patients
Source: Biomed Res Int. 2017 Oct 15;2017:9849823. doi: 10.1155/2017/9849823 (PMC5661091; doi:10.1155/2017/9849823)
Supplement: Supplementary file 1 — Primers used for amplifying in our study. [file 9849823.f1.doc]

**Supplemental Tables**

Table 1 Primers used for amplifying the core region and NS5B region

| Gene fragments | Primers | | 5'-3' sequence |
| --- | --- | --- | --- |
| **Core region** | Outer primers | Forward | 5′-ACTGCCTG ATAGGGTGCTTGC-3′ |
|  |  | Reverse | 5′-ATGTACCCCAT GAGGTCGGC-3′ |
|  | Inner primers | Forward | 5'-AGG TCTCGTAGACCGTGCA-3' |
|  |  | Reverse | 5'-CATGTGAG GGTATCGATGAC-3' |
| **NS5B region** | Outer primers | Forward | 5'-CNTAYGGITTCCARTACTCICC-3' |
|  |  | Reverse | 5'-GAG GARCAIGATGTTATIARCTC-3' |
|  | Inner primers | Forward | 5'-TATGAYACCCGCTGYTTTGACTC-3' |
|  |  | Reverse | 5'-GCNGARTAYCTVGTCATAGCCTC-3' |

**Table 2　Primers used for HCV NS3 amplification by Nested PCR**

| HCV genotype | Primer | 5’-3’Sequence |
| --- | --- | --- |
| 6a | up1 | 5′-TAGCGACGGACTGCGCGATC-3′ |
|  | down1 | 5′-GAGTGCCACTCCACAGACCC-3′ |
|  | up2 | 5′-GCTCCCATCACCGCGTATGC-3′ |
|  | down2 | 5′-GTCAGGACTGTAACCACAGG-3′ |

**Table 3 Primers used for HCV NS5A amplification by Nested PCR**

| HCV genotype | Primer | 5’­­-3’Sequence |
| --- | --- | --- |
| 6a | up1 | 5′-TCACCAACGCACTACGTGCC-3′ |
|  | down1 | 5′-TTGCAAGCCCTTGCTCAGGG-3′ |
|  | up2 | 5′-GTGCCTGAGACTGACGCGTC-3′ |
|  | down2 | AGGTCGATGGCGTGAGGATA-3′ |

**Table 4 Primers used for HCV NS5B amplification** by Nested PCR

| HCV genotype | Primer | 5’­­-3’Sequence |
| --- | --- | --- |
| 6a | Section1up1 | 5′-TCATGGTCGACGGTCAGTAG-3′ |
|  | Section1down1 | 5′-AGTGTAACACCAATAGACAC-3′ |
|  | Section1up2 | 5′-GGCCGACACGGAAGATGTCG-3′ |
|  | Section1down2 | 5′-GTGTGGAAAGACCTTCTGGA-3′ |
|  | Section2up1  Section2down1  Section2down1  Section2down1 | 5′-GGCGTGCGCGTGTGCGAGAA-3′  5′-GACTTGTCCGGTTGGTTCAC-3′  5′-GTACGACGTGGTTAGCAAGC-3′  5′-AGGGCTGCCATATGTGGCAA-3′ |
|  | Section3up1 | 5′-TTGGATGAGTCCACGGTCTC-3′ |
|  | Section3down1 | 5′-TTGGGCAACATCATTATGTT-3′ |
|  | Section3up2 | 5′-GATCCAGGACCTAGTTCAGA-3′ |
|  | Section3down2 | 5′-ATTACTACCTCACACGTGAC-3′ |
